# Horizontal inequity in the use and access to health care in Uruguay

**DOI:** 10.1186/s12939-020-01237-w

**Published:** 2020-10-26

**Authors:** Cecilia González, Patricia Triunfo

**Affiliations:** Departamento de Economía, Facultad de Ciencias Sociales, Universidad de la República, Montevideo, Uruguay

**Keywords:** Health inequality, Concentration index, Uruguay, I100, I180, I190, Desigualdad en salud, Índices de concentración, Uruguay, I100, I180, I190

## Abstract

**Background:**

In 2007 Uruguay began a reform in the health sector towards the construction of a National Integrated Health System (SNIS), based on public insurance with private and public provision. The main objective of the reform was to universalize access to health services.

**Methods:**

Data comes from the first National Health Survey conducted in 2014 and available since 2016. Concentration indices are calculated for different indicators of use and access to medical services, for the population 18 years of age and older, and for different subgroups (age, sex, region and type of coverage). The indices are decomposed into need and non-need variables and the contribution of each of them to total inequality is analyzed. Horizontal inequity is calculated.

**Results:**

Results show pro-rich inequality for medical consultations, medical analysis, medication use and non-access due to costs. Type of health coverage is the variable that explains most of the inequality: private coverage is pro-rich while public coverage is pro-poor. Income does not appear as significant to explain inequality, except for access issues.

From the population subgroups’ analysis, there is no evidence of inequality for the group of 60 years old or more. On the other hand, studies such as Pap Smear and prostate, which may be associated with preventive studies,, shows pro-rich inequality and, in both cases, the main contribution is given by income.

**Conclusions:**

The analysis of health inequity shows pro-rich inequity in medical consultations, medical analysis, medication use and lack of access due to costs. The type of health coverage explains these inequalities; in particular, private coverage is pro-rich. These results suggest that the type of health coverage are capturing the income factor, since higher income individuals will be more likely to be treated in the private system.

## Resumen

**Antecedentes:**

En 2007 el gobierno uruguayo comenzó una reforma del sector salud tendiente a la construcción de un Sistema Nacional Integrado de Salud, basado en un seguro público con provisión privada y pública. El principal objetivo de la reforma fue universalizar el acceso a los servicios de salud.

**Metodología:**

A partir de la primera Encuesta Nacional de Salud realizada en 2014 y disponible en 2016, se calculan índices de concentración para diversos indicadores de uso y acceso a servicios médicos, para la población de 18 años y más, y para diferentes subgrupos (edad, sexo, región y tipo de cobertura), a la vez que se descomponen los índices en variables de necesidad y no necesidad, analizando la contribución de cada una de dichas variables a la desigualdad total, y calculando la inequidad horizontal.

**Resultados:**

Los resultados muestran inequidad horizontal pro rico para las consultas médicas, análisis médicos, uso de medicamentos y falta de acceso por motivo de costos. El tipo de cobertura de salud es la variable que más explica la desigualdad: la cobertura privada es pro rico mientras que la cobertura pública es pro pobre. El ingreso no resulta significativo para explicar la desigualdad excepto para los problemas de acceso.

Del análisis de subgrupos de población se destaca que no se encuentra evidencia de inequidad para el grupo de 60 años y más. Por otro lado, estudios como el PAP y de próstata, que pueden ser asociados a conductas preventivas, muestran inequidad pro rico, y en ambos casos la principal contribución está dada por el ingreso.

**Conclusiones:**

El análisis de la inequidad en salud muestra inequidad pro rico en consultas médicas, análisis médicos, uso de medicamentos y problemas de acceso por motivo de costos. El tipo de cobertura de salud explica en gran parte estas desigualdades, en particular, la cobertura privada es pro rico. Estos resultados sugieren que el tipo de cobertura médica estaría captando el factor ingreso, ya que los individuos de mayores ingresos tienen mayor probabilidad de atenderse en el sistema privado.

## Background

Uruguay is a small country, with an ageing population relative to the region and a long tradition of social protection, public education and health. Currently, total health expenditure represents about 10% of the GDP, 70% of which is public expenditure.

In 2007 the government began a reform in the health sector towards the construction of a National Health Integrated System (SNIS, for its acronym in Spanish), based on public insurance (FONASA for its acronym in Spanish) with public or private provision. All workers and pensioners contribute to FONASA, and as a counterpart, are entitled to choose a comprehensive health care, either from the network of public clinics and hospitals administered by the State Health Care Administration, or from a Collective Medical Care Institution (private institutions). The main objective of the reform was to universalize access to health services, setting a schedule for the gradual expansion of FONASA to different groups, which finished in 2016 (dependents of formal workers, children and spouses, retirees, etc.).

In this type of systems, with public insurance and co-payments limited by the regulator, access barriers (waiting times, logistical difficulties of access, etc.) are used as a rationing mechanism. In this context, it is interesting to analyze how the use and access to health services are distributed by socioeconomic level in Uruguay. Moreover, this is particularly relevant if we take into account that there are no previous studies considering total population, not even after the health system reform.

Therefore, the main objective of this paper is to analyze horizontal equity in the Uruguayan health systems. Horizontal equity implies that individuals with the same health care needs will receive the same care, regardless of their level of income or other characteristics such as sex, education, etc. On the other hand, vertical equity denotes unequal access to health care for people with different needs.

Several studies for the Organization for Economic Co-operation and Development (OECD) countries find that the poorest groups are more likely to make a visit to the general practitioner, although horizontal inequity is not detected in most of European countries analyzed. In contrast, visits to specialists are concentrated in high income groups [16–21]. Despite differences in the coverage system’s between countries, studies show a decrease in inequality over the years.

A recent study that considers 18 OECD countries and data from 2000 to 2006, finds that pro-rich inequity in medical consultations, particularly specialists, dentists and preventive care, still remain in most countries, although in different magnitudes [7]. These results show great inequity in the United States and France. Particularly, the first presents the highest inequity in terms of visits to the doctor and the dentist, while France shows the highest inequity in terms of visits to specialists and studies to detect cancer. On the other hand, Switzerland and the United Kingdom have the lowest levels of inequity.

Focusing on Latin America, Suárez-Berenguela [15] follow the same methodology as the studies mentioned above and analyze inequity in health status and access to medical care in several Latin American countries. The results show that inequality is more substantial in access to medical care than in health status, and in turn in preventive studies rather than in curative care.

More recent studies, such as Vásquez et al. [22] for Chile, found that pro-rich inequity for visits to specialists and dentists, and pro-poor inequity for general practitioner visits, emergency room visits and days of hospitalization, improved in all cases between 2000 and 2009. The largest contribution to pro-rich inequity is given by private health coverage and education, while the greatest contribution to pro-poor inequity is given by income and education.

In the case of Brazil, Almeida et al. [2] found that the use of medical and dental services is pro-rich, although inequity had been decreasing between 1998 and 2008. Factors that contribute most to inequity are private health coverage, education and income.

As for Mexico, Barraza-Lloréns et al. [5] show that curative visits (not preventive) and hospitalizations are more concentrated in the richest population, and no significant changes were found between 2000 and 2006. Health insurance, education and socioeconomic status are the aspects that contribute the most to the inequitable distribution of health care.

Furthermore, Granda and Jimenez [9] found that health inequality decline after the public health system reform in Ecuador. However, curative visits show a pro-rich bias, and the use of public health facilities is concentrated among the poor. Income, family size and education are the most relevant determinants of inequality in health care utilization. However, after the reform, the impact of income in the utilization of curative visits decreased.

In the case of Uruguay, the only previous work is Balsa et al. [3, 4] which analyzes the horizontal inequity in access to medical care for the elderly in Montevideo using a specific health survey. Authors find horizontal inequity in the quality of access to medical visits in favor of those from higher socioeconomic status.

Previous empirical evidence suggests that differences between countries may be affected by different health coverage systems (universal or not, types of financing, co-payments or not, etc.), by different types of systems organization (for example: need or not of a general practitioner visit to access consultation with medical specialists), by type of provision (private or public), etc.

In this regard, it is important to provide evidence for Uruguay after the reform of the health system began in 2008. The aim of this work is to measure, for the first time, the horizontal inequity in the use and access to medical care for the entire Uruguayan population. Data comes from the first National Health Survey conducted by the Ministry of Public Health in 2014 (and available in 2016).^1^ Monitoring this type of information is essential to determine if the policy objectives established in the reform, such as equity in access, are being met.

## Methodology

We estimated concentration indices (CI) [11, 12] that allow the measuring of the degree of socioeconomic inequality present in use and access to health services [13, 23–25] .

The *CI*_*m*_, where *m* is the indicator of health services, varies between − 1 and 1 (when the poorest or the richest individual receives all medical care, respectively), and a value of 0 means absence of inequality.

When the indicator of use of health services, *m*_*i*_, is a binary variable, an alternative approach consist in estimating it through a linear approximation to the non-linear model [13]. A detail of this approach is presented in Appendix 1.

CI can be decomposed in order to quantify the contribution of different categories of factors to inequality. Van Doorslaer et al. [21] proposes the decomposition of the CI in need variables, non-need variables and socioeconomic variables. This decomposition allows to appreciate how total inequality is affected by the different variables. In this sense, the CI can be expressed as the weighted sum of inequality in each of its determinants [13, 21]. Each specific index is weighted by the elasticity of the use of health care regarding each determinant. Following Fleurbaey and Schokkaert [8], the inequality of non-need determinants is the component associated with legitimate inequity, in the sense that individual behavior does not contribute to good health and may require a more intensive use of medical care. Horizontal inequity, *HI*, represents inequality in the use of care that is not justified by inequalities in morbidity or medical care needs [10].^2^ It can be obtained by subtracting the contribution of need variables from total inequality.

In this article, HI is estimated for different indicators of use and access to medical care. Estimations were made for the general population (18 years and over) and by age groups, sex, type of medical coverage and region (Montevideo and rest of the country).^3^

## Data

Data comes from the first National Health Survey (ENS for its acronym in Spanish), carried out in Uruguay in 2014 by the Ministry of Public Health.^4^ ENS was conducted for individuals living in households in areas with 5000 or more inhabitants, all around the country. The survey included 4096 individuals, collecting information from the household and from one selected person, inquiring about their health status, habits, health expenditure, and socioeconomic characteristics (sex, age, education and income). This study only considered individuals 18 years old or more years old.

Health care utilization variables include physician visits (binary variable equal to 1 if the person visited a general practitioner, a specialist, or an emergency room in the last 30 days), medical exams (binary variable equal to 1 if the person made laboratory analysis, ultrasounds, eco Doppler, etc. in the last 30 days), and use of medications (binary variable equal to 1 if the person used any medications in the last 30 days). Two preventive medical exams were distinguished: the PAP Smear (binary variable equal to 1 for women older than 21 years old who declared getting the PAP Smear in the last 3 years) and prostate examination (binary variable equal to 1 for man older than 40 years old who declared getting the study at least once in a lifetime). Mammography was not considered since very few women reported getting it during the last 2 years. Finally, two variables related with problems of access to health care services were defined: non-access due to cost-problems (binary variable equal to 1 if the person needed some kind of medical attention or medical exam and could not get it due to lack of money, no money for copayments, etc. in the last 12 months) and non-access by logistic problems (binary variable equal to 1 when the problem was related to distance to the hospital, delays to schedule medical visits or exams).

The income variable used was the household’s per capita income. Income variable in the ENS has two problems. First, an underreported income level regarding to the Continuous Household Survey^5^ (ECH for its acronym in Spanish) was found for the same year. Second, a 10% of non-response was found in the household income variable. Taking this into account, imputation of income was made using data from the ECH. In a first stage, regressions of the household income logarithm in the ECH was estimated for eight subgroups using age and sex, on a set of variables correlated with the socioeconomic status and replicable in the ENS. All regressions had an R2 of 0.6 or greater. In a second stage, the logarithm of household income in the ENS was predicted using the coefficients of the first estimate. This procedure was made for all the cases in the ENS, not only in the cases of “non-responses”. Per capita household income was calculated using the square root of the number of household members.^6^

The variables associated with health care needs are age, sex, and self-reported health status. The ENS’s variables used as indicators of the health status were the self-report of the general state of health, the prevalence of chronic non-communicable diseases, and the presence of functional limitations. For self-reported health status, a variable that is worth 1 is defined when the person declares to have an excellent or very good state of health; it is worth 2 when the person declares to have a good health status, and it is worth 3 when the person declares to have a regular or bad state of health.^7^ In turn, the variable of chronic non-communicable diseases is worth 1 when the person has at least one of these diseases or when they have biological risk factors that can cause them.^8^ Last but not least, the physical limitations variable takes the value 1 if the person suffers at least one limitation.^9^

Finally, non-need variables considered were personal habits, consumption of tobacco or alcohol, sedentary lifestyle, eating habits and medical coverage.^10^ Regarding the latter, several binary variables are specified: private coverage by FONASA, which takes the value 1 if the person declares to have health coverage in private institutions through the National Health Insurance; public coverage by FONASA, which takes the value 1 if the person declares to have health coverage in public institutions through the National Health Insurance; private coverage, which takes the value 1 if the person declares to have health coverage in private institutions but is not a beneficiary of the of the National Health Insurance; and public coverage, which takes the value 1 if the person declares to have health coverage in public institutions but is not a beneficiary of the of the National Health Insurance or if that person declares not to have coverage. The latter is the reference category.

After eliminating cases of double health coverage,^11^ the final sample considered corresponds to 3814 cases. The descriptive statistics of the main variables used are presented in Table 1. As for age, 49% of the sample is between 18 and 44 years old, 20% is between 45 and 59 years old and 31% is 60 years old and older. Men are 44% of the sample. Some highlights include: 62% declare to have an unhealthy diet, 17% are sedentary and 23% smoke every day. In addition, 56% claim to have some chronic non-communicable disease and 12% have some physical limitation. Regarding health coverage, 52% have private coverage through FONASA and 32% have public coverage without FONASA. About the use of health services, 33% attended a medical consult in the last 30 days and 11% had a medical exam done, while 70% used medication. Regarding preventive exams, 40% of women over 21 years old got a PAP Smear in the last 3 years, while 47% of men over 40 years of age got the prostate exam once.

**Table 1:** Descriptive statistic, population 18 years of age or older, 2014

	Percentage	Confidence intervals (95%)	
**Age**			
18–44	0.487	0.4715	0.5033
45–59	0.198	0.1853	0.2106
60 +	0.315	0.2999	0.3294
**Men**	0.438	0.4218	0.4533
**Health coverage**			
Not coverage	0.018	0.0136	0.0220
Private coverage by FONASA	0.517	0.4980	0.5298
Public coverage by FONASA	0.077	0.0686	0.0856
Private coverage	0.099	0.0889	0.1078
Public coverage	0.307	0.2926	0.3219
**Personal habits:**			
Unhealthy diet	0.618	0.6023	0.6332
Sedentary life	0.168	0.1549	0.1803
Alcohol	0.099	0.0897	0.1101
Tobacco	0.232	0.2175	0.2461
**Health status**			
Chronic noncommunicable diseases	0.563	0.5474	0.5789
Physical limitations	0.118	0.1075	0.1280
**Health care utilization**			
Physician visits (30 days)	0.333	0.3183	0.3482
Medical exams (30 days)	0.112	0.1024	0.1225
Use of medications (30 days)	0.709	0.6943	0.7231
Non-access by cost-problems (12 months)	0.091	0.0818	0.1001
Non-access by logistic-problems (12 months)	0.126	0.1153	0.1364
PAP Smear (3 years)	0.397 (a)	0.3738	0.4193
Prostate examination (ever)	0.471 (b)	0.4373	0.5039
N total sample	3814		
Person expansion factor	675.154	657.3001	693.0086

Source: Own elaboration based on ENS (MSP, 2014)

Note: All variables are dummies variables

(a) Women over 21 years of age (1783 observations)

(b) Men over 40 years of age (867 observations)

## Results

Table 2 shows the estimates of the concentration indices (CIm) for the different categories of use and access to medical care, for the population 18 years of age or older. All estimates were weighted using an expansion factor (the average weight of Stata Corp.) considering the weight of the person. This weight is the inverse of the probability of the inclusion of the person (obtained from the probability of housing selection dividing by the number of people in the household without including children under 1 year of age).^12^

**Table 2:**
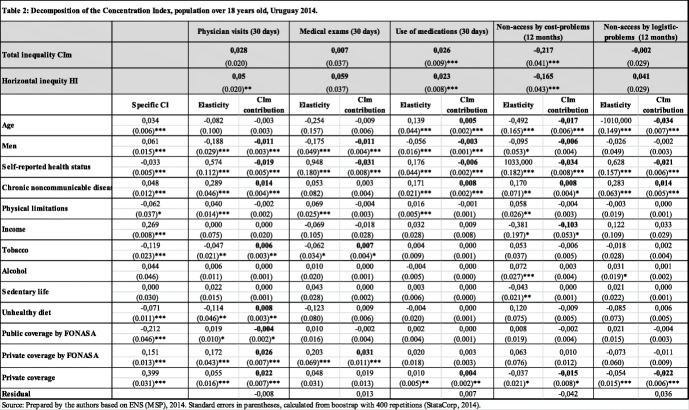
Decomposition of the Concentration Index, population over 18 years old, Uruguay 2014

Source: Prepared by the authors based on ENS (MSP), 2014. Standard errors in parentheses, calculated from boostrap with 400 repetitions (StataCorp, 2014)

Note: all estimates weighted with the personal expansion factor (aweight command in STATA)

The contribution to inequality of each of the variables of need and non-need is also presented. This contribution is determined by the distribution of this variable in relation to income (specific CI) and by the elasticity of the use of care in relation to the some variable. Need variables considered are age, sex (men), health status (self-reported), suffering from chronic non-communicable diseases (NCDs) and having some physical limitation. Non-need variables considered are individuals’ habits (smoke and alcohol consumption, unhealthy eating, sedentary), health coverage (public through FONASA, private through FONASA or private) and income. Finally, Table 2 shows estimates of horizontal inequity, calculated as the difference between the CIm and the need-factors’ contribution to inequality.

Results show that the CIm for the entire population is significant in cases of use of medications and non-access to medical care due to economic reasons, showing the use of medications is concentrated among rich people and the non-access problems are concentrated among lower income individuals. The horizontal inequity index is significant and positive for physician visits and use of medication, and negative for non-access by cost problems, suggesting a pro-rich distribution.

Regarding the contribution of the different factors to inequality, need-variables, when significant, have a negative contribution (pro-poor). This is the case for variables men, health status and physical limitations. This contribution is determined by the specific CI of the factor and by the elasticity of use. For example, the self-reported health status has a pro-poor contribution to inequality, determined by a negative specific CI and by a positive elasticity of use. The first indicates that the distribution of those who reported worse health status is concentrated among individuals of lower socioeconomic status while the second indicates greater use among those who reported worse health status.

Contribution of non-need variables, when significant, show that bad health habits have a pro-rich contribution to inequality since they reflect the socioeconomic characteristics of the groups in which these habits are concentrated. In particular, the distribution of those who smoke or have an unhealthy diet is concentrated among lower income individuals, while the negative elasticity of use indicates less use by this group of individuals.

Private health coverage (FONASA or other), when significant, contributes to the increase in inequality (pro-rich) while public coverage by FONASA contributes to reducing it (pro-poor). This is the case for physician visits and exams, the use of medication and the non-access. It is relevant to point out that income contribution to inequality is not significant.

A possible explanation could be that health coverage variables are capturing the income factor, since individuals with higher incomes are more likely to be treated through the private health system. Indeed, it can be observed that public coverage has a negative CI, indicating a higher concentration of this variable among the poorest individuals. In contrast, the private coverage variables have a positive CI, indicating that private health coverage is concentrated among the richest individuals.

**Fig. 1:**
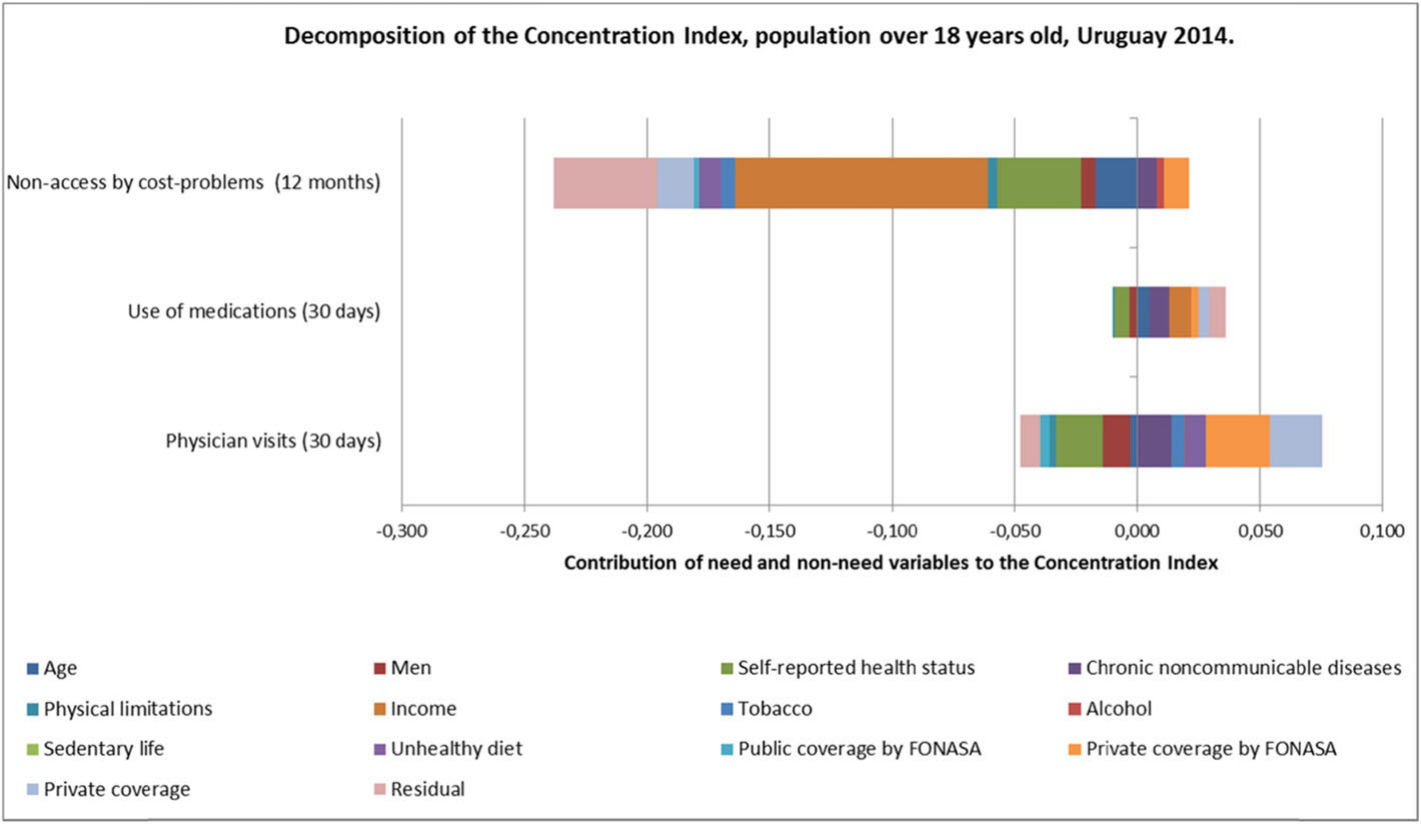
Decomposition of the Concenration Index, population over 18 years old, Uruguay 2014

Figure 1 presents the results of decomposition analyses for the statistically significant variables. The CI decomposition indicates the contributions of different variables to inequality.

In Appendix 2 we presented the Lorenz Curves for variables with significant CI (Physician visits, Use of medications, Non-access by cost-problems). In the case of Non-access by cost problem, the concentration curve lies above the line of equality. That means this problem is concentrated among the poor. These curves allow to visualize more clearly the results found when calculating the inequality indices. On the one hand, that although there is pro-poor inequality in these indicators, the concentration is low in relation to other Latin American countries (see for example Vásquez et al. [22] for the case of Chile; Almeida et al. [2] for Brazil, or Barraza (2013) for Mexico. On the other hand, the curves allow us to easily visualize the greatest inequalities found in non-access due to costs.

Table 3 shows the CI_m_ and its decomposition for different groups of population. Need variables’ contribution is presented in column 2. Non-need variables’ contributions are disaggregated in columns 3 to 7 in order to visualize the impact of different types of health coverage. Column 9 shows horizontal inequity.

**Table 3:**
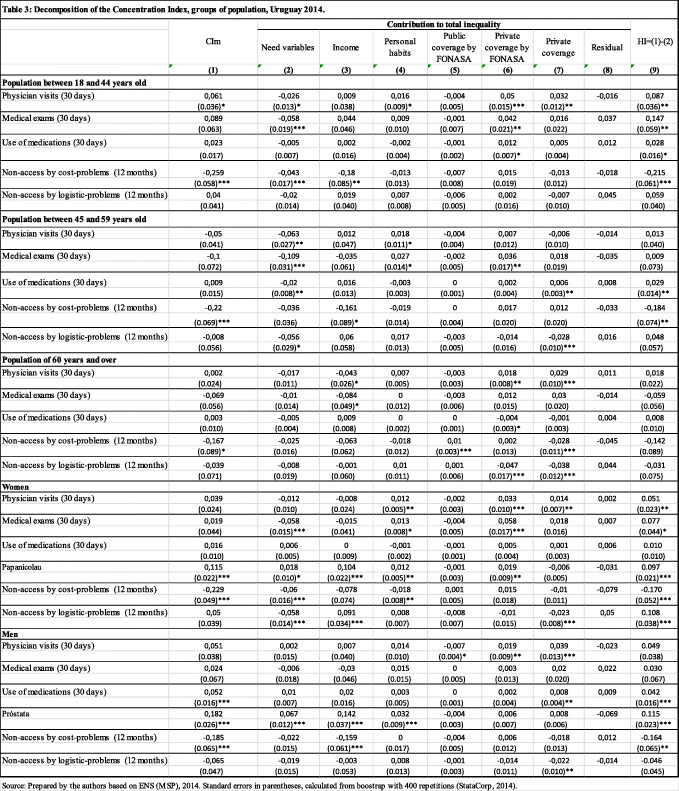
Decomposition of the Concentration Index, groups of population, Uruguay 2014

Source: Prepared by the authors based on ENS (MSP), 2014. Standard errors in parentheses, calculated from boostrap with 400 repetitions (StataCorp, 2014)

Note: all estimates weighted with the personal expansion factor (aweight command in STATA)

Regarding age, population between 18 and 44 years of age encounters a pro-rich inequality in the physician visits and in non-access due to costs. The main contributions to inequality are given by private health coverage (with or without FONASA) and by income (in the case of non-access due to costs). The contribution of need variables is pro-poor (except for non-access due to costs), indicating that health care needs are concentrated among individuals of lower socioeconomic status who use different health services. When need variables are not taken into account, the resulting HI is significant for all the indicators and pro rich (except for the non-access by logistic problems). As for individuals between 45 and 59 years old, pro-rich inequality is observed in non-access due to costs and income has a pro-poor contribution The HI is significant and pro rich, and also for the use of medication.

There is no evidence of inequality for population of 60 years of age and older. Solely in the case of non-access due to costs, the CI_m_ is significant and pro-rich. The greatest contribution to pro-rich inequality comes from health coverage. Need variables do not make a significant contribution. At the same time, for this age group it is not possible to reject the hypothesis of horizontal equity in any of the variables of use and access to medical services. Balsa et al. [3] in a previous work estimated concentration indices and horizontal inequity for adults over 60 years of age residing in Montevideo, capital of Uruguay. They found pro-rich inequity in access to physician visits. Since this study was conducted before the health system reform, it is interesting to compare its results with those from the ENS. For this purpose, estimates were made by selecting a sample from the ENS with the same characteristics (over 60 years of age and residents in Montevideo). None of the CI_m_ or HI obtained were statistically significant.^13^ However, these results are not conclusive as an evaluation of the health reform. Firstly, the selected cases from the ENS were very few (only 394 cases) since the sample was not specifically designed for that age group. Secondly, at the time when the ENS took place, not all older adults had been incorporated into the new health system.

Regarding the analysis for women and men, it is worth noting that results in relation to specific preventive studies by sex. In the case of women, there is pro-rich inequality in the procurement of the PAP Smear. The main contribution to inequality is income. This result is striking since PAP Smear and mammography exams have been exempted from co-payments, within the framework of health promotion policies according to Ministry of Public Health guidelines, since 2006. The HI index is significant and pro-rich for all indicators, except in the use of medications. In the case of men, there is pro-rich inequality in the use of medication, the prostate exam and in non-access due to costs. The main contribution to inequality is income (pro-rich). As for the prostate exam, income contribution is followed by the contribution of need variables that are also pro-rich. The HI index is significant and pro rich in these three cases.

Moreover, results by region show inequality in the use of medications and non-access by cost-problems for both regions considered. The country’s capital city show pro-rich HI for the variables considered (excepted non-access by logistic problems), while the rest of the country only shows inequity in non-access due to costs (Table 4).

**Table 4:**
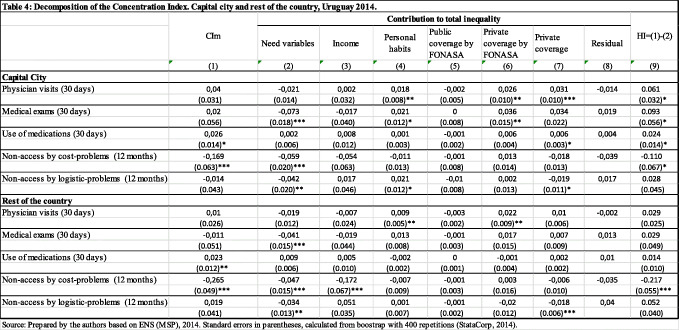
Decomposition of the Concentration Index. Capital city and rest of the country, Uruguay 2014

Source: Prepared by the authors based on ENS (MSP), 2014. Standard errors in parentheses, calculated from boostrap with 400 repetitions (StataCorp, 2014)

Note: all estimates weighted with the personal expansion factor (aweight command in STATA)

Finally, regarding the analysis related to health coverage (Table 5), results are presented for those who have private coverage by FONASA and public coverage without FONASA, since the other two types of coverage do not have enough cases to carry out the analysis. Among private system’s users with FONASA coverage, stands out the presence of inequality in non-access due to cost. The main contribution to inequality is income. Also, a pro-rich HI is observed. Among the public system’s users, stands out the inequality in the use of medications, income is the variable that contributes the most to that inequality, and HI is pro-rich.

**Table 5:**
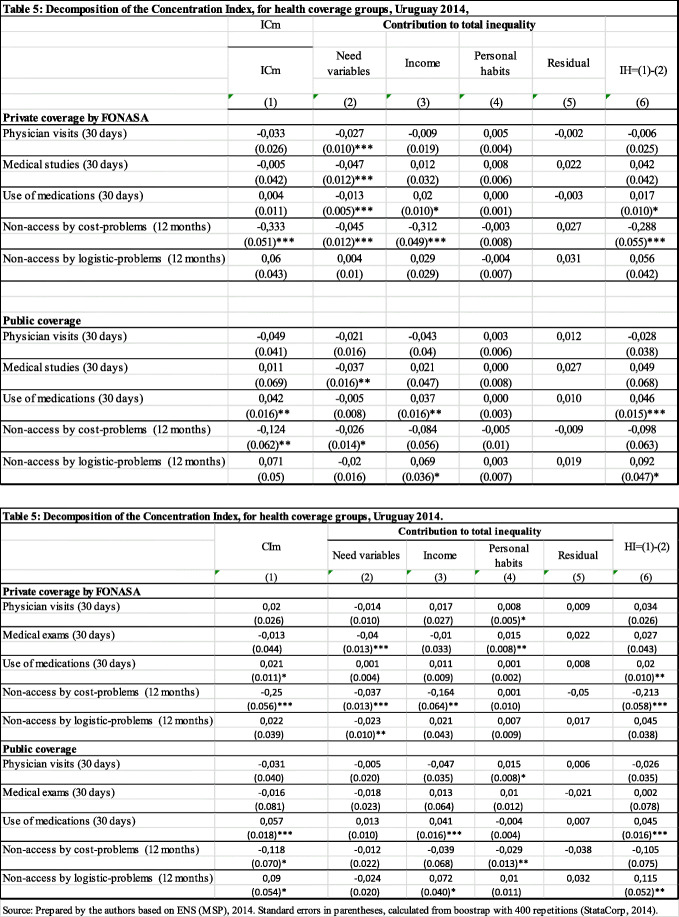
Decomposition of the Concentration Index, for health coverage groups, Uruguay 2014

Source: Prepared by the authors based on ENS (MSP), 2014. Standard errors in parentheses, calculated from boostrap with 400 repetitions (StataCorp, 2014)

Note: all estimates weighted with the personal expansion factor (aweight command in STATA)

Some final considerations about the methodology need to the made. As it has already been mentioned, health care variables are binary, so it is advisable to use nonlinear models. Nevertheless, a linear approximation to the nonlinear model was calculated for all estimates since this allows the decomposition analysis. In addition, calculating concentration indices of binary variables has generated a debate regarding the possibility of comparing results between countries. For this reason, a comparative analysis with other countries in the region is not attempted. Another aspect to take into consideration is that this analysis does not control the potential endogeneity between the need for health services and the medical care received, which may be due to the contemporary measurement of both variables. Moreover, nor does it control for potential endogeneity between income and the use of health care, which could be due to the simultaneity in the measurement or due to the existence of omitted need variables that affect the use of the services and may be correlated with income.

## Conclusions

This work analyzes the existence of horizontal inequity in access to health care for the entire population over 18 years of age. Data used comes from the First National Health Survey conducted in Uruguay in 2014. Concentration indices were calculated for various indicators of use and access to medical services in Uruguay for the population over the age of 18, and for different subgroups. Also, a decomposition of these indices into factors of necessity and not necessity was performed. Finally, the existence of horizontal inequity was analyzed. The National Health Survey was conducted after the health system reform carried out in 2008. Therefore, there is no available data from before the reform to compare possible effects on inequity and access to health care. Nevertheless, this work has an interest in itself since it is the first time that horizontal inequity is studied for the whole adult population throughout the country.

Results show the presence of inequality in the case of non-access problems to health services due to cost. The above includes non-access to physician visits, exams, surgical interventions and other treatments due to the cost of co-payments, transfers, etc. Index decomposition shows income is the main contributor to this inequality.

However, there is evidence of horizontal inequity in physician visits, use of medication and non-access by cost-problems.

It is interesting to note that, when significant, health coverage plays an important role to explain inequality. Private coverage leads to increasing inequality (pro-rich), while public coverage helps to reduce it. A possible explanation for this is that health coverage variables are capturing the effect of the income factor, since individuals with higher incomes are more likely to be treated in the private system, and even more so in the private sector without FONASA.

The analysis centered in different subgroups of the population shows that inequality in non-access due to costs is present in all groups considered (divided by age, sex, region and type of health coverage). The greatest contribution to inequality is given by income in all cases. Another noteworthy result is that there is no evidence of horizontal inequity for the group of 60 years of age and older. On the other hand, the procurement of medical exams by sex, that could be associated with preventive behavior (PAP Smear in the case of women and prostate exam in the case of men), shows evidence of inequality and pro-rich inequity, and in both cases the main contribution to inequality is given by income. Finally, when comparing geographical location, it can be observed that the capital city of the country presents evidence of inequality and inequity in a greater number of variables of use and access than the rest of the country.

## Figures and Tables

**Fig. 2:**
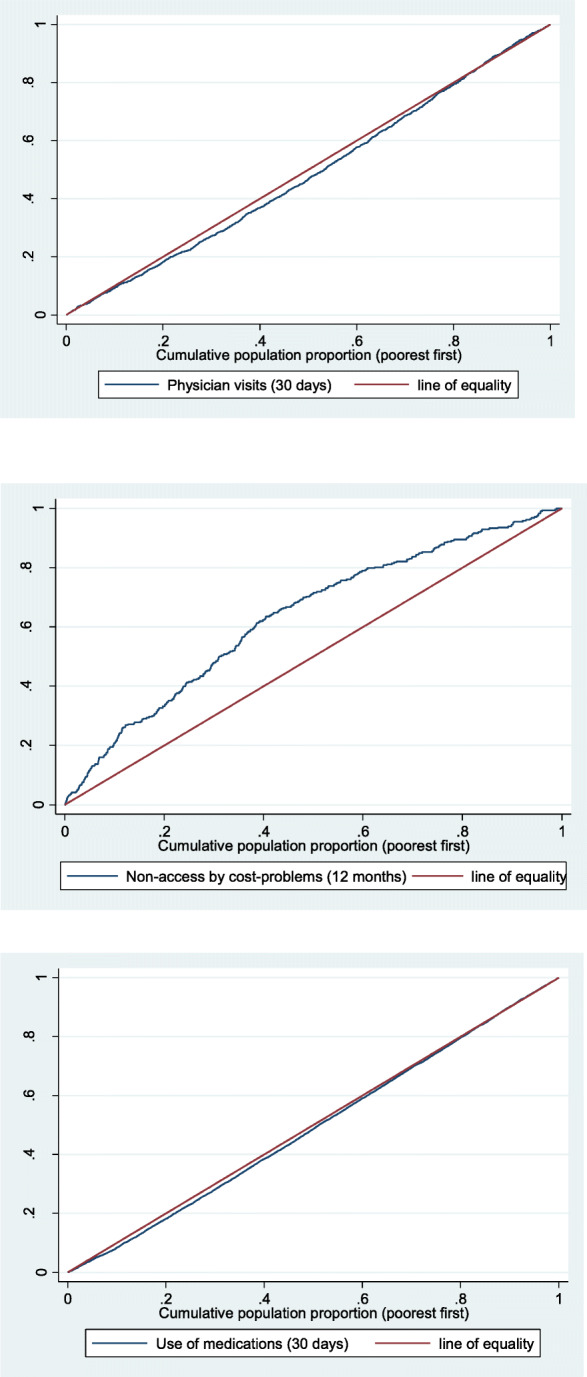
Concentration curves for health care utilization

## Data Availability

Datasets used and analyzed during the current study are available from the corresponding author on request.

## Appendix 1

The most generalized approach to measure the magnitude of horizontal inequity has been through concentration indices [10, 11]. These concentration indices (CI) allow the measuring of the degree of socioeconomic inequality present in a variable, particularly in variables of use and access to health services ([21, 23], Wagstaff and van Doorslaer, [22])).^14^ They are defined as follows:

1 $$ {CI}_m=\frac{2}{N\overline{m}}{\sum}_{i=1}^N\left({m}_i-\overline{m}\right)\left({R}_i-1/2\right), $$

where *m*_*i*_ is the indicator of health services’ use for the individual *i*, *N* is the sample size, $\overline{m}$ is the average utilization of the health services, and *R*_*i*_ is the cumulative proportion of the population ordered by the socioeconomic variable of interest (income or deprivation) until the individual *i*. The *CI*_*m*_ varies between − 1 and 1 (when the poorest or the richest individual receives all medical care, respectively), and a value of 0 means absence of inequality.

When the indicator of use of health services, *m*_*i*_, is a binary variable, an alternative approach consist in estimating it through a linear approximation to the non-linear model, as follows [12]^15^:

2 $$ {m}_i={\alpha}_0^m+{\alpha}_1^m{y}_i+{\sum}_k{\beta}_k^m{h}_{ik}+{\sum}_j{\gamma}_j^m{x}_{ij}+{u}_i, $$

where *β*^*m*^, *γ*^*m*^ and ${\alpha}_1^m$ are the partial effects of the non-linear model’s variables, treated as fixed parameters and evaluated in the mean of the sample; *u*_*i*_ is the error term, which includes approach errors; *y*_*i*_ represents the income or socioeconomic status; *h*_*i*_ = (*h*_*i*1_, …., *h*_*iK*_) includes need variables, and *x*_*i*_ = (*x*_*i*1_, …. *x*_*ij*_, ) includes non-need variables.

CI can be decomposed in order to quantify the contribution of different categories of factors to inequality. Van Doorslaer et al. [19] proposes the decomposition of the CI in need variables, non-need variables and socioeconomic variables, combining Eqs. 1 and 2). This decomposition allows to appreciate how total inequality is affected by the different variables.

In this sense, the CI can be expressed as the weighted sum of inequality in each of its determinants [12, 19]:

3 $$ {CI}_m=\left({\alpha}_1^m\overline{y}/\overline{m}\right){CI}_y+{\sum}_k\left({\beta}_k^m{\overline{h}}_k/\overline{m}\right){CI}_{h_k}+{\sum}_j\left({\gamma}_j^m{\overline{x}}_j/\overline{m}\right){CI}_{x_j}+{GCI}_u/\overline{m}, $$

where *CI*_*y*_, *CI*_*hk*_, *CI*_*xj*_ are concentration indices that measure inequality in income distribution, in care needs and in non-need variables respectively, and *GCI* is the generalized concentration index for the error term. Each index is weighted by the elasticity of the use of health care regarding each determinant. Following Fleurbaey and Schokkaert [7], *CI*_*x*_ is the component associated with legitimate inequity, in the sense that individual behavior does not contribute to good health and may require a more intensive use of medical care. Horizontal inequity, *HI*, represents inequality in the use of care that is not justified by inequalities in morbidity or medical care needs [9].^16^ It can be obtained by subtracting the contribution of need variables from total inequality in the previous equation. In this way, it can be obtained that:

4 $$ HI={CI}_m-{\sum}_k\left({\beta}_k^m{\overline{h}}_k/\overline{m}\right){CI}_{h_k}, $$

In this article, HI is estimated for different indicators of use and access to medical care. Estimations were made for the general population (18 years and over) and by age groups, sex, type of medical coverage and region (Montevideo and rest of the country).^17^

## Abbreviations

CI: Concentration Indices
ENS: Encuesta Nacional de Salud (National Health Survey)
ECH: Encuesta Continua de Hogares (Household Survey)
FONASA: Fondo Nacional de Salud (National Health Insurance)
HI: Horizontal inequity
MSP: Ministerio de Salud Pública (Ministry of Public Health)
PAP: Papanicolaou test (PAP Smear)
SNIS: Sistema Nacional Integrado de Salud (National Integrated Health System).

## Footnotes

1 http://www.msp.gub.uy/sites/default/files/archivos_adjuntos/Primer%20Informe%20Encuesta%20Nacional%20de%20Salud%20%282016%29.pdf

2 This index does not control for potential endogeneity between the needs for health services and the medical care received, which may be due, for example, to the contemporary measurement of both variables; neither between income and the use of health services, due to both the simultaneity in the measurement and the existence of omitted need-variables that affect the use of services and may be correlated with income [3, 4]. Only works that use longitudinal data overcome these problems

3 Standard errors are calculated through bootstrapping with 400 replications (StataCorp, 2014).

4 The ENS was based on a randomized, stratified, and multi-stage sample design (three stages in Montevideo and four stages in the rest of the country), The sampling frame was the 2011 Census. More details in “First Report of National Health Survey (2016)” in https://www.gub.uy/ministerio-salud-publica/datos-y-estadisticas/datos/encuesta-nacional-de-salud

5 Continuous Household Survey is a national survey with a long tradition of confiability, performed by the National Statistics Institute. Income information from this Survey is using to calculate official statistics about poverty and income inequality.

6 As a robustness check, another strategy for imputation of income was carried out. The method performs multiple stochastic imputations, using information from the variable itself as well as the relationship between different ENS variables. This method assumes that the probability that the data is missing does not depend on unobservable characteristics (they are “missing at random”). The missing data was imputed 10 times to give variability of the imputation. An algorithm was used based on the so-called chained equations (Stata software), which performs a sequence of univariate imputations with specific models for each variable (“Full 6 Conditional Specifications”). Therefore, a multivariate imputation is carried out, using the conditional density of the observed data for each variable. The results do not vary significantly from those presented in this paper and are available by request.

7 The five original options of health status were grouped into three categories due to the low frequency of some of these options.

8 Diseases considered were renal failure, heart problems, hyper or hypothyroidism, respiratory diseases (emphysema or asthma), bone-muscle diseases (arthritis / osteoarthritis, rheumatism, osteoporosis, tendinitis and spinal problems); biological risk factors considered were overweight or obesity, hypertension, high cholesterol and diabetes.

9 Limitations considered were: blindness, difficulties to see even with glasses, deafness, hearing difficulties even using hearing aids, speech difficulties, inability or difficulty to move that requires the permanent use of a wheelchair, impossibility or difficulty to move that requires the use of a cane or crutches permanently, limitations for using hands and arms, limitations for moving outside the house or using means of transport, limitations for moving inside the house, mental limitations that make learning and application of knowledge and task development difficult; mental limitations that make it difficult to interact with others.

10 The ENS allows for a partial approximation to these behaviors, since it does not release information necessary to establish the risk levels. In this sense, the binary variables are defined: alcohol, which takes the value 1 if the person consumed alcohol three or more times per week, or if he got drunk several times, in the last 30 days; sedentary, which takes the value 1 if the person declares that he is sitting for most of the day and at the same time does not usually perform at least 10 minutes of physical activity; smoke, takes the value 1 if the person currently smokes every day. As for eating habits, the ENS collects the frequency of habitual consumption of a set of foods (not the amount consumed), allowing to specify the binary variable unhealthy food that takes the value 1 if the person consumes fruits or vegetables 1 or 2 times per week (or less), if eats snacks (chips, cookies), “fast” food or drinks with sugar 3 times per week (or more).

11 Double coverage refers to people who declare have public and private health coverage.

12 More details in “First Report of National Health Survey (2016)” in https://www.gub.uy/ministerio-salud-publica/datos-y-estadisticas/datos/encuesta-nacional-de-salud

13 The results are available by request.
